# Li_3_V_2_(PO_4_)_3_ Cathode Material: Synthesis Method, High Lithium Diffusion Coefficient and Magnetic Inhomogeneity

**DOI:** 10.3390/ijms25052884

**Published:** 2024-03-01

**Authors:** Tatiana Gavrilova, Yulia Deeva, Anastasiya Uporova, Tatiana Chupakhina, Ivan Yatsyk, Alexey Rogov, Mikhail Cherosov, Ruslan Batulin, Mikhail Khrizanforov, Sergey Khantimerov

**Affiliations:** 1 Kazan E. K. Zavoisky Physical-Technical Institute, FRC Kazan Scientific Center of RAS, Sibirsky Tract, 10/7, 420029 Kazan, Russia; i.yatzyk@gmail.com (I.Y.); alexeyrogov111@gmail.com (A.R.); khantim@mail.ru (S.K.); 2 Institute of Solid State Chemistry of the Ural Branch of RAS, Pervomaiskaya Str., 91, 620990 Ekaterinburg, Russia; juliahik@mail.ru (Y.D.); nastyauporova99@gmail.com (A.U.); chupakhina@yandex.ru (T.C.); 3 Institute of Physics, Kazan Federal University, Kremlyovskaya Str., 18, 420008 Kazan, Russia; mcherosov@gmail.com (M.C.); tokamak@yandex.ru (R.B.); 4 Arbuzov Institute of Organic and Physical Chemistry, FRC Kazan Scientific Center of RAS, Arbuzov Str., 8, 420088 Kazan, Russia; khrizanforov@gmail.com; 5 Aleksander Butlerov Institute of Chemistry, Kazan Federal University, 1/29 Lobachevskogo Str., 420008 Kazan, Russia

**Keywords:** Li_3_V_2_(PO_4_)_3_, cathode material, discharge capacity, lithium diffusion coefficient, electron spin resonance, magnetization, magnetic inhomogeneity

## Abstract

Li_3_V_2_(PO_4_)_3_ cathodes for Li-ion batteries (LIBs) were synthesized using a hydrothermal method with the subsequent annealing in an argon atmosphere to achieve optimal properties. The X-ray diffraction analysis confirmed the material’s single-phase nature, while the scanning electron microscopy revealed a granular structure, indicating a uniform particle size distribution, beneficial for electrochemical performance. Magnetometry and electron spin resonance studies were conducted to investigate the magnetic properties, confirming the presence of the relatively low concentration and highly uniform distribution of tetravalent vanadium ions (V^4+^), which indicated low lithium deficiency values in the original structure and a high degree of magnetic homogeneity in the sample, an essential factor for consistent electrochemical behavior. For this pure phase Li_3_V_2_(PO_4_)_3_ sample, devoid of any impurities such as carbon or salts, extensive electrochemical property testing was performed. These tests resulted in the experimental discovery of a remarkably high lithium diffusion coefficient D = 1.07 × 10^−10^ cm^2^/s, indicating excellent ionic conductivity, and demonstrated impressive stability of the material with sustained performance over 1000 charge–discharge cycles. Additionally, relithiated Li_3_V_2_(PO_4_)_3_ (after multiple electrochemical cycling) samples were investigated using scanning electron microscopy, magnetometry and electron spin resonance methods to determine the extent of degradation. The combination of high lithium diffusion coefficients, a low degradation rate and remarkable cycling stability positions this Li_3_V_2_(PO_4_)_3_ material as a promising candidate for advanced energy storage applications.

## 1. Introduction

Energy is a fundamental driver in the development and progression of human society [1,2]. In recent times, there has been a growing recognition of the limitations and environmental impact of traditional fossil fuels [3]. Renewable energy sources, while environmentally friendly, present challenges due to their dependence on geographical and climatic conditions. One of the main challenges is how to meet peak demand for renewables. Solar and wind generation, for instance, are dependent on weather conditions, leading to variability in energy production. Efficient and reliable storage solutions are needed for the energy and transportation industries to address this challenge. Energy storage technology can be broadly separated into electrical, thermal and fuel technologies, with batteries, fuel cells and supercapacitors being the main storage solutions for renewable energy generation [4]. Electrical storage systems, in particular, have seen significant advancements and innovations over the past decade. The focus has been predominantly on developing more efficient and reliable batteries and capacitors, which are critical for a variety of applications ranging from portable electronic devices to large-scale energy storage for power grids [5,6,7].

Lithium-ion batteries (LIBs) emerged as a leader in this field, primarily due to their high specific energy density, which significantly surpasses that of traditional battery technologies [8]. This high energy density makes LIBs an attractive option for a wide range of applications, from powering small electronic devices to serving as the primary energy storage method in electric vehicles [9]. Moreover, ongoing research and development in LIBs technology are continuously enhancing their efficiency, lifespan and safety, making them even more appealing for both current and future applications. In recent years, the quest for more sustainable and efficient energy solutions has brought significant focus to the development of advanced materials for LIBs [10,11,12].

One of the most promising avenues in this field is the exploration and enhancement of cathode materials, which play a crucial role in elevating the overall performance, energy density and durability of LIBs. Among various candidates, lithium vanadium phosphate Li_3_V_2_(PO_4_)_3_ (LVPO) emerged as a material of interest due to its impressive electrochemical properties. The study and creation of efficient LVPO cathode materials are driven by the need to address some of the critical challenges faced by current LIBs technologies, including limited energy density, reduced cycle life and issues related to safety and environmental impact [13,14,15]. LVPO, with its unique crystal structure and electrochemical stability, presents an opportunity to overcome these obstacles, offering a pathway to higher energy densities and enhanced safety features. The interest in LVPO stems from its high theoretical capacity combined with its high lithium diffusion coefficient, which promises significant improvements over traditional cathode materials like lithium cobalt oxide (LiCoO_2_) and lithium iron phosphate (LiFePO_4_) [16,17,18,19,20,21,22]. Moreover, LVPO is known for its excellent thermal stability, a critical factor in preventing thermal runaway and ensuring safer battery operations [23]. This aspect is particularly important in the context of increasing demand for electric vehicles (EVs) and large-scale energy storage systems, where safety and reliability are paramount.

Another compelling attribute of LVPO is its lower environmental impact compared to other cathode materials that contain cobalt or nickel. The use of vanadium, a more abundant and less toxic element, aligns with the growing emphasis on environmentally sustainable battery technologies. Furthermore, LVPO’s ability to operate over a wide range of temperatures enhances its suitability for diverse applications, ranging from portable electronics to grid storage. The development of LVPO cathode materials also includes addressing challenges such as optimizing the synthesis process, enhancing the electronic and ionic conductivity and ensuring compatibility with other battery components. Research into doping strategies, novel synthesis techniques and surface modifications is vital in realizing the full potential of LVPO.

In this article, we delve into the various aspects of LVPO cathodes, exploring their composition, structural advantages, electrochemical performance and the latest advancements in their development. For example, it is well known that the kinetics of lithium-ion transport are of significant importance for electrode materials. Lithium diffusion in the electrodes is a key factor that determines the rate at which a battery can be charged and discharged. In most cases, the solid-state diffusion of Li ions with rather low values may control the rate-determining step of the intercalation process, although there may be particular cases where the rate-determining step is diffusion in the electrolyte solution within the pores of the composite electrodes. One of the main advantages of LVPO is the potentially higher rate of Li^+^ ion transport in the structure: the LVPO electrodes show higher lithium diffusion coefficients compared to that of traditional cathode materials like LiCoO_2_ and LiFePO_4_ (up to 10^−9^ cm^2^ s^−1^) [24,25]. However, diffusion coefficients for LVPO are in the range of 10^−13^ to 10^−9^ cm^2^ s^−1^, depending on many factors, including the synthesis method and additives [26,27]. Thus, the development of electrode material based on LVPO with a higher rate of Li^+^ ion transport in the structure is of great interest, and the successful synthesis of such material is described in this article.

In addition to X-ray diffraction analysis and scanning electron microscopy methods, the successfully synthesized LVPO was examined using electron spin resonance and magnetometry methods to quantify defects, determine their distribution state, what could be the reason for the promising performance of LVPO as a cathode material in lithium-ion batteries. It is known from the literature that exploring the intrinsic material properties from a magnetic perspective offers a unique angle for electrochemical investigations [28]. Considering the intimate connection between spin and magnetic properties, using electron spin as a probe, magnetic measurements (nuclear magnetic resonance, electron spin resonance, magnetometry and Mössbauer spectroscopy) make it possible to analyze energy storage processes from the perspective of spin and magnetism [28]. Nuclear magnetic resonance (NMR) has attracted much attention in the field of energy storage because of its ability to provide information on reversible and irreversible transient changes in composition, ion transport dynamics and microstructure evolution in an electrochemical cell [29,30]. Electron spin resonance (ESR) detects the qualitative and quantitative information of unpaired electrons contained in local atoms or molecules of matter and the structural characteristics of its surrounding environment. NMR is commonly used in the study of light elements such as Li, Na, O and F, while ESR is suitable for the evaluation of transition metals (Fe, Co, Mn, V) and oxygen vacancies and for the characterization of redox processes [13,31,32,33]. Owing to the ultrahigh sensitivity to magnetization, magnetometry can accurately monitor the magnetic changes caused by electron transfer during electrochemical processes, and it is a powerful tool for investigating conversion or alloy-type alkali metal ion batteries (AMIBs), spin-based devices [34,35] or solid electrolyte interphase characterization by operando magnetometry [36].

Here, we aim to provide a comprehensive understanding of how LVPO stands to revolutionize the field of lithium-ion batteries and pave the way for more sustainable, efficient and safer energy storage solutions from the point of view of the relationship between electrochemical and magnetic properties. The characterization of the synthesized sample using comprehensive techniques, including X-ray diffraction analysis, scanning electron microscopy, magnetometry and electron spin resonance methods, is given. Extensive electrochemical property investigations for the detailed characterized material were conducted, including lithium diffusion coefficient estimations and cycling stability testing. Additionally, relithiated LVPO (after multiple electrochemical cycling) samples were investigated to determine the extent of degradation, showcasing the material’s potential for long-term applications in Li-ion batteries.

## 2. Experimental Results

### 2.1. Characterization of As-Prepared Li_3_V_2_(PO_4_)_3_ Sample

#### 2.1.1. Structural and Microstructural Properties

**Figure 1:**
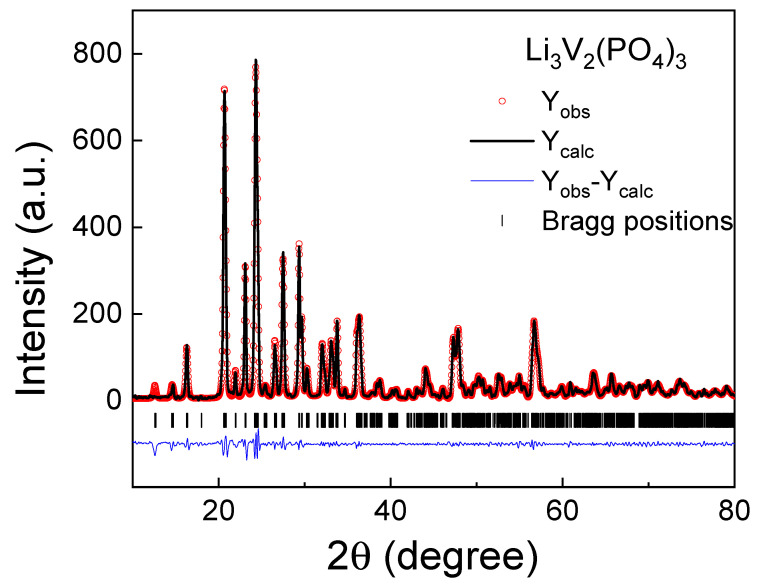
Experimental, theoretical and differential X-ray diffraction pattern of Li_3_V_2_(PO_4_)_3._

The X-ray diffraction pattern of the LVPO sample investigated in this work is presented in Figure 1. According to X-ray diffraction data, the resulting product is a single-phase LVPO sample with a monoclinic crystal structure (space group *P*2_1_/*n* (#14)). The refined crystal structure parameters of a synthesized LVPO sample are *a* = 8.6027(3) Å, *b* = 8.5908(1) Å, *c* = 12.0363(5) Å, β = 90.58(3)°, and the cell volume is V = 889.505(4) Å^3^. The reliability factors of the Rietveld refinement are as follows: R_p_ = 3.78%, R_wp_ = 6.9%, χ^2^ = 0.567. The unit cell contains Z = 4 formula units. The obtained lattice parameters are very close to those that were previously reported and can slightly differ depending on the synthesis process and monoclinic axis selection [13,32,33,37,38,39,40,41,42]. The structure of LVPO consists of a three-dimensional framework of slightly distorted VO_6_ octahedra and PO_4_ tetrahedra. The alkali metal ions (Li-ions) occupy interstitial sites and can have different oxygen environments; thus, the unit cell contains three independent lithium sites. The LVPO crystal structure visualization is well represented in the literature, for example, as shown in Figure 1 of Ref. [32].

**Figure 2:**
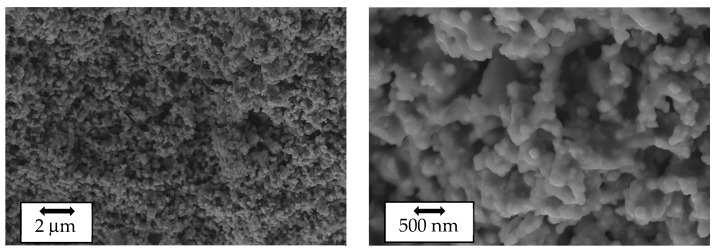
Scanning electron microscope images of the as-prepared Li_3_V_2_(PO_4_)_3_ sample at different magnifications.

SEM images of the as-prepared LVPO surface are shown in Figure 2. The SEM images reveal that the as-prepared composites exhibit a granular structure with average grain sizes of a few micrometers. The element-selective images obtained using XRF analysis are shown in Figure S4, confirming the uniform distribution of elements.

#### 2.1.2. Magnetic Properties

**Figure 3:**
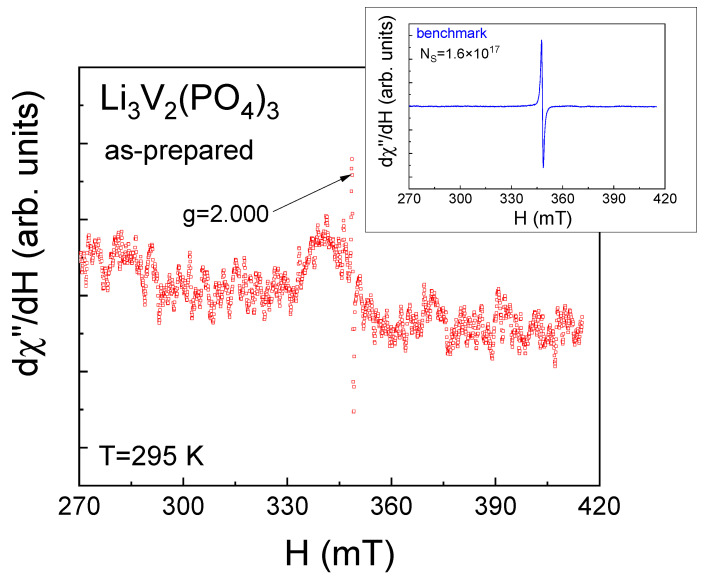
Electron resonance spectrum of as-prepared Li_3_V_2_(PO_4_)_3_ at room temperature at the X-band frequency. Inset shows the electron spin resonance spectrum of the benchmark containing N_s_ = 1.6∙× 10^17^ spins.

It can be assumed that the observed ESR spectra (Figure 3) are likely due to the presence of magnetic vanadium ions in the investigated LVPO sample, specifically due to a small number of V^4+^ ions (electronic configuration 3d^1^, S = 1/2). V^3+^ ions (electronic configuration 3d^2^, S = 1) that form the LVPO crystal structure are non-Kramer’s ions with integral J in LS coupling, which have an even number of electrons in the respective electronic shells, singlet ground-state levels and are silent under conventional ESR experimental conditions [43,44]. To observe ESR signals from the exited (S = 1) state of V^3+^ ions due to the transitions between sublevels with Δm_S_ = 1, this state should be populated at a given temperature by heating or laser irradiation, etc. The second condition for ESR detection from the exited (S = 1) state in the conventional perpendicular state is that the energy of the microwave field should be sufficient for the transition between these sublevels. Based on a set of experimental data given in the literature [31,45,46] and our previous works [13,32,33], no signal is expected from trivalent vanadium in perpendicular mode for the above reasons, which may exist separately or together. The direct probe of V^3+^ via the ESR test was presented in [31]. C. Li et al. observed forbidden transitions with Δm_S_ = 2 for V^3+^ in parallel mode in the related compound Na_3_V_2_(PO_4_)_2_O_1.6_F_1.4_, while in parallel mode, the ESR signal from V^3+^ was not observed [31,46].

V^5+^ ions (electronic configuration 3d^0^) are nonmagnetic and ESR-silent. The ex situ continuous-wave (CW) X-band electron spin resonance measurements of carbon-coated LVPO nanocomposites allowed for the investigation of the evolution of the valence state of vanadium ions upon cycling, indicating the appearance of the V^3+^/V^4+^ redox couple or the oxidation of almost all V^3+^ ions up to V^4+^ state [45].

The presence or absence of tetravalent vanadium ions is important for the following reason. In an ideal stoichiometric LVPO compound, all vanadium ions should be in the trivalent state. The change in the valence state of vanadium ions from V^3+^ to V^4+^ in LVPO can be associated with lithium non-stoichiometry in the investigated compound. To maintain the electrochemical neutrality of the unit cell, the change in the valence of one vanadium ion from 3+ to 4+ corresponds to the deintercalation of one lithium-ion. Considering the vanadium-to-lithium ratio in the chemical formula, it is possible to estimate the degree of lithium nonstoichiometry in the investigated samples.

The ESR method was used to estimate the number of V^4+^ ions. To achieve this, the integral intensity of the LVPO spectrum was compared with the same parameters for the benchmark (inset Figure 3). The ESR spectra integral intensities ratio of the investigated samples (I_LVPO_) and the benchmark (I_0_) are given in Line 2 of Table 1. The corresponding number of V^4+^ magnetic centers in the investigated samples can be estimated as N(V^4+^) = I_LVPO/LPO_/I_0_·N_s_ (Line 3 in Table 1), where N_s_ is the spin number in the benchmark. The total number of vanadium ions, N_0_, is given in Line 4 of Table 1. The relative number of tetravalent vanadium ions is shown in Line 5 of Table 1. It can be seen from Table 1 that the as-prepared LVPO sample demonstrates high lithium stoichiometry.

In addition to electron spin resonance measurements, the magnetic properties of LVPO were investigated using the magnetometry method. This was performed to determine whether a small number of mixed valence vanadium ions create magnetic inhomogeneity. Based on our previous experiences, we know that a small amount of mixed-valence transition element ions can create magnetic inhomogeneity [47,48,49] that can directly affect other physical properties [47,48].

**Figure 4:**
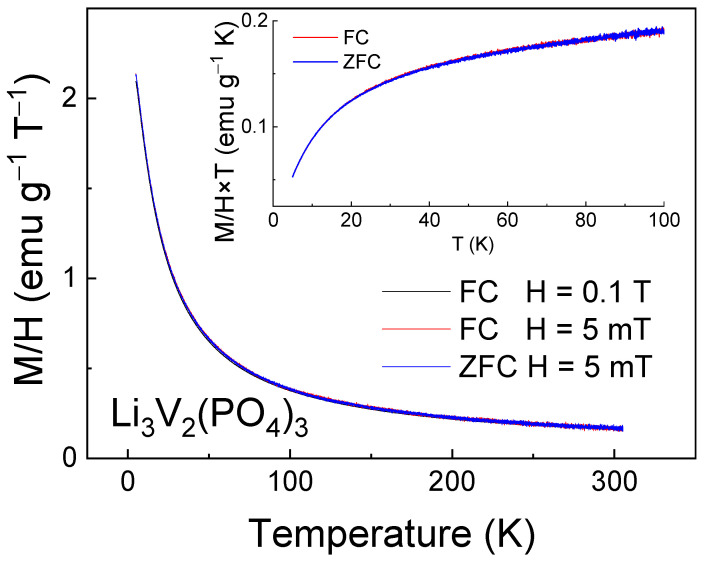
Magnetization M/H as a function of temperature for Li_3_V_2_(PO_4_)_3_ sample measured in the FC regime in the external magnetic field H = 0.1 T. Insets show the low temperature data in representation M × T vs. T in more detail.

Magnetization measurements in the ZFC-FC regimes in low magnetic fields (Figure 4) were performed to determine the ZFC-FC splitting temperature below which the magnetic correlations due to the presence of vanadium ions of mixed valence become dominant over thermal fluctuations. This type of splitting was not observed in LVPO, suggesting the absence of significant short-range magnetic correlations in the investigated sample. Nevertheless, the decrease in the product M∙T (which is proportional to the squared effective magnetic moment) with decreasing temperature suggests that antiferromagnetic (AFM) interactions are present (inset in Figure 4).

**Figure 5:**
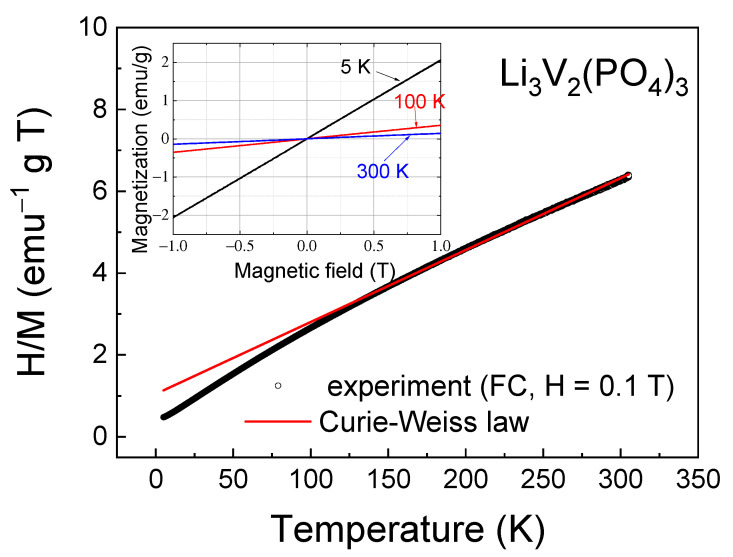
Inverse magnetic susceptibility H/M as a function of temperature; solid line corresponds to the Curie–Weiss law (see details in the text). Inset shows the magnetization for Li_3_V_2_(PO_4_)_3_ as a function of the external magnetic field at different temperatures.

To estimate the value of AFM interactions, the inverse magnetic susceptibility H/M was fitted by the Curie–Weiss law χ = *C*/(T − θ_CW_) in its inverse form χ^−1^ = (T − θ_CW_)*/C*, where *C* is the Curie constant and θ_CW_ is the Curie–Weiss temperature (Figure 5). This was performed at temperatures above T > 120 K, where the inverse magnetic susceptibility is linear. The high-temperature approximation of the experimental data by the Curie–Weiss law gives negative values of the Curie–Weiss temperature θ_CW_ = −59.5 K, confirming the antiferromagnetic nature of the exchange interactions between spins in the investigated sample.

The higher absolute value of the Curie–Weiss temperature in LVPO compared to LVPO/C composite [32] indicates stronger magnetic interactions in pure LVPO. However, when comparing with Li_3_V_2_(PO_4_)_3_ (92.5 wt.%)/Li_3_PO_4_ (7.5 wt.%) composite, one can see close values of the Curie–Weiss temperature: θ_CW_ = −68 K for composite [33] and θ_CW_ = −59.5 K for LVPO, and therefore, close values of AFM interactions for both samples. The comparison of Curie–Weiss temperature and Curie constant values with the same values for Li_3_V_2_(PO_4_)_3_ (86 wt.%)/Li_3_PO_4_ (14 wt.%) composite seems difficult due to the small linear range in the temperature dependence of the inverse magnetic susceptibility to approximate by the Curie–Weiss law (Figure S5). The deviation from the Curie–Weiss law in the Li_3_V_2_(PO_4_)_3_ (86 wt.%)/Li_3_PO_4_ (14 wt.%) composite is due to the presence of a significant number of magnetically correlated regions [13].

Despite the high values of the Curie–Weiss temperature, AFM interactions are not sufficient for short-range and, especially, long-range magnetic orders to arise. This suggestion can be confirmed by isothermal magnetization measurements as a function of the external magnetic field (inset in Figure 5), which showed that in the investigated field range up to H = 1 T, the M-H curves were linear without any tendency towards saturation and hysteresis.

The approximation of the inverse dependencies of the H/M curve (Figure 5) yields a Curie constant value of *C* = 56.9 emu∙K∙g^−1^∙T^−1^ = 2.32 emu∙K∙mol^−1^∙Oe^−1^, in addition to the Curie–Weiss temperature. The effective magnetic moment can be calculated from the Curie constant as follows:

(1) $$\mu_{eff}=\sqrt{3k_{B}C/N_{A}}$$

where *k_B_* is the Boltzmann constant, *C* is the Curie constant and *N_A_* is Avogadro’s constant. The obtained *μ_eff_* for LVPO is 4.31 *μ_B_*. Considering that the magnetic ion V^3+^ has a 3d^2^ electronic configuration and a ground state ^3^F with spin *S* = 1, we can estimate the effective magnetic moment *μ_eff_* as

(2) $$\mu_{theor}=g\cdot \sqrt{Z\cdot S\cdot (S+1)}\cdot \mu_{B}$$

where *μ_B_* is the Bohr magneton, *g* is the Lande g-factor, *Z* is the number of magnetic ions in a unit cell and *S* is the spin. Taking into account that *g* = 1.95 [32] for vanadium ions, we obtain a theoretical effective magnetic moment per mole of *μ_theor_* = 3.9 *μ_B_*. The theoretical and experimental values of the effective magnetic moment for the LVPO composite coincide within a 10% error margin. This discrepancy could be attributed to the approximation error due to the insignificant linear section in the temperature dependence of reverse magnetization (Figure 5). There are no other apparent reasons for the experimentally obtained absolute value of the effective magnetic moment to exceed the theoretical one unless it is due to the presence of magnetic ordering.

### 2.2. Electrochemical Performance

**Figure 6:**
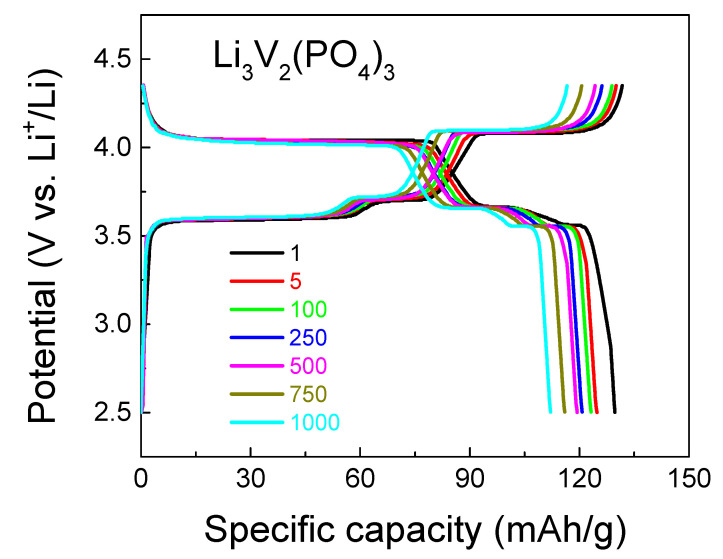
Charge–discharge characteristics of the Li_3_V_2_(PO_4_)_3_ cathode material for various cycle numbers (black—1 cycle, red—5 cycles; green—100 cycles; blue—250 cycles; pink—500 cycles; beige—750 cycles; cyan—1000 cycles) at a 1C rate. The equivalence in mA/g for the LVPO cathode material is 126 mAh/g.

**Figure 7:**
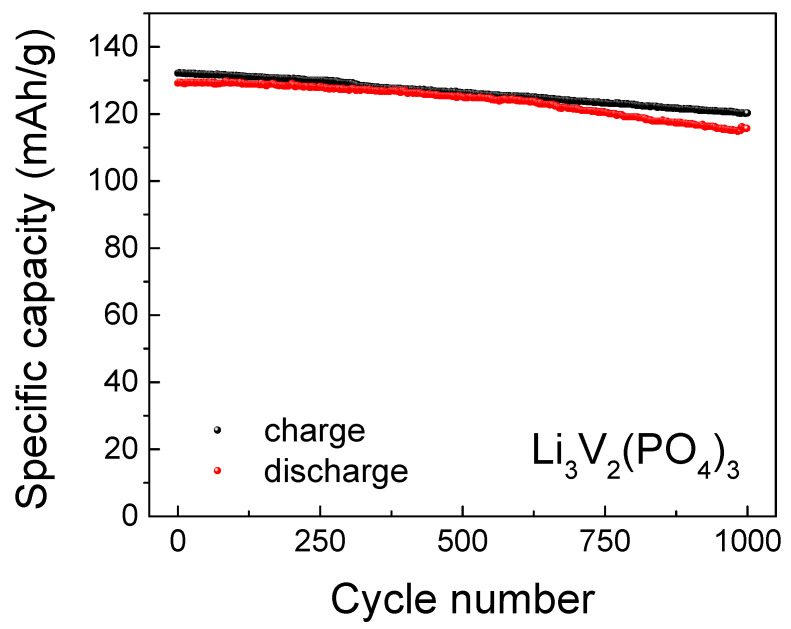
Long-term cycle performance at 1C up to 1000 cycles in the voltage window 2.0–4.5 V for Li_3_V_2_(PO_4_)_3_ sample.

**Figure 8:**
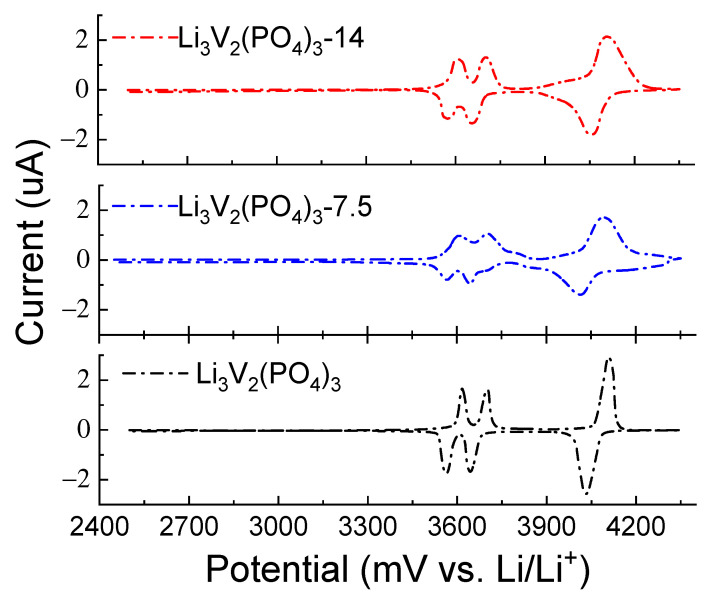
Cyclic voltammograms of cathode material samples Li_3_V_2_(PO_4_)_3_–LVPO (black), Li_3_V_2_(PO_4_)_3_ (92.5 wt.%)/Li_3_PO_4_ (7.5 wt.%)–LVPO/LPO-7.5 (blue) and Li_3_V_2_(PO_4_)_3_ (86 wt.%)/Li_3_PO_4_ (14 wt.%)–LVPO/LPO-14 (red), at a temperature of 25 °C.

Figure 6 and Figure 7 display the charge–discharge characteristics (charge–discharge capacity depending on the number of charge–discharge cycles) and long-term cycle performance at 1C, the equivalence in mA/g for the LVPO cathode material is 126 mAh/g, respectively. With regard to the cyclic voltammograms of three types of vanadates, Li_3_V_2_(PO_4_)_3_ cathode material samples are compared with the same data for the previously reported Li_3_V_2_(PO_4_)_3_ (86 wt.%)/Li_3_PO_4_ (14 wt.%) and Li_3_V_2_(PO_4_)_3_ (92.5 wt.%)/Li_3_PO_4_ (7.5 wt.%) composites [13,33] at a scan rate of 0.5 mV s^−1^, as presented in Figure 8. The composites Li_3_V_2_(PO_4_)_3_ (86 wt.%)/Li_3_PO_4_ (14 wt.%) and Li_3_V_2_(PO_4_)_3_ (92.5 wt.%)/Li_3_PO_4_ (7.5 wt.%) are labeled as LVPO/LPO-14 and LVPO/LPO-7.5, respectively. This approach provides a more consistent and reliable means to evaluate the diffusion kinetics of lithium ions in various electrode compositions. The CV method’s ability to capture rapid changes in electrochemical behavior at different scan rates makes it particularly suitable for this type of analysis, offering detailed insights into the lithium-ion mobility within the electrode structure.

**Figure 9:**
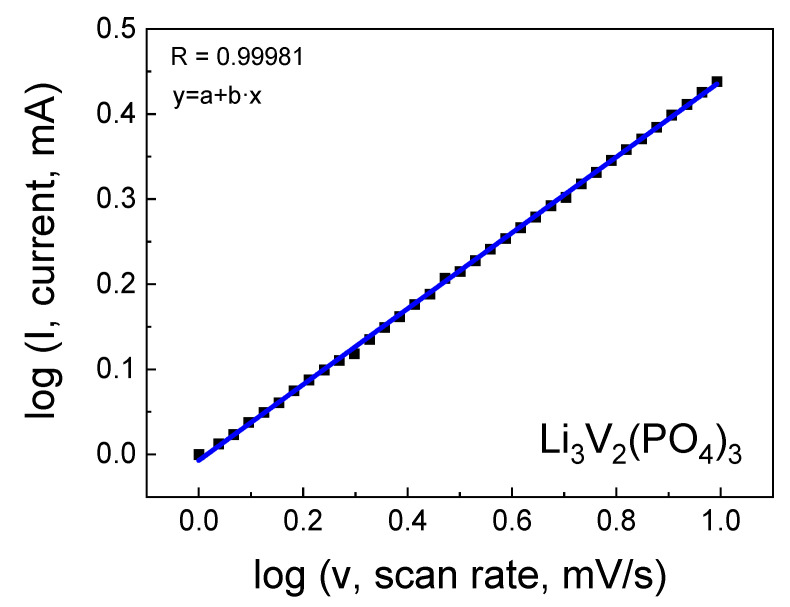
Logarithmic dependence of current on scan rate.

In cyclic voltammetry analysis, it is evident that the presence of salt in the sample leads to the broadening of the wave and alteration of its amplitude, indicating a change in the lithium diffusion coefficient. As the scan rate increases, the peaks on the CV gradually broaden while retaining reversibility with slight overpotentials at different scan rates. An analysis was conducted to study the relationship between the scan rate and peak current, as shown in Figure 9. The results demonstrated that the (de)intercalation of ions for the electrode is governed by diffusion processes. With an increase in scan rate from 0.1 to 0.5 mV s^−1^, the capacitive contribution accounted for 98.1%, suggesting a throughput facilitated by a combination of rapid reaction kinetics and high performance.

From the results of the linear approximation of cyclic voltammetry, the corresponding slope was calculated, followed by the determination of the lithium-ion diffusion coefficient (*D*) using Equation (3), which was found to be 2.82 × 10^−12^ cm^2^/s and 2.69 × 10^−11^ cm^2^/s for the LVPO-7.5 and LVPO-14 samples, respectively. Differences in the lithium-ion diffusion coefficients in these samples could be attributed to variations in structure, particle size and the amount of impurity, which impact diffusion properties. It is noteworthy that for the pure phase Li_3_V_2_(PO_4_)_3_ with high lithium stoichiometry, the value of the lithium-ion diffusion coefficient is significantly higher. In our studies, it was found that the pure LVPO exhibited a notably high lithium diffusion coefficient of *D* = 1.07 × 10^−10^ cm^2^/s. This value is significantly higher than those reported for other LVPO composites and variants, highlighting the superior ionic conductivity of the pure LVPO material. The high diffusion coefficient of pure LVPO can be attributed to its well-ordered crystal structure, which facilitates the smooth passage of lithium ions during the charge and discharge cycles. This structural advantage results in enhanced electrochemical performance, characterized by rapid charge–discharge capabilities and the improved overall energy efficiency of the battery.

Moreover, the high diffusion coefficient indicates that pure LVPO can effectively accommodate rapid electrochemical processes, making it a highly suitable material for applications requiring high power densities, such as in electric vehicles and high-power electronics. This property, combined with the inherent thermal stability and safety of LVPO, positions it as an attractive candidate for next-generation lithium-ion batteries.

In conclusion, the discovery of a high lithium diffusion coefficient in pure LVPO underscores its potential as a high-performance cathode material. It opens up avenues for further research and development in optimizing LVPO-based batteries for various high-demand applications, promising advancements in the field of energy storage technologies.

### 2.3. Sample Characterization after Intercalation/Deintercalation Process

**Figure 10:**
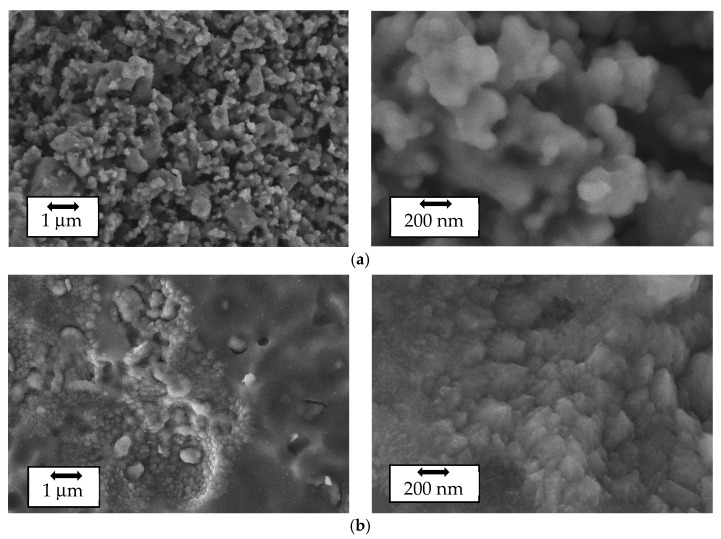
SEM images of Li_3_V_2_(PO_4_)_3_ surfaces after multiple charge/discharge cycles (100 cycles) at different magnifications: (**a**) relithiated sample, (**b**) delithiated sample.

In addition to the as-prepared sample, the morphology of Li_3_V_2_(PO_4_)_3_ was investigated during the lithium intercalation/deintercalation process. SEM images of multiple relithiated samples (at the lithiation and delithiation stages) of Li_3_V_2_(PO_4_)_3_ are shown in Figure 10. The elemental composition and elemental mapping were investigated by the X-ray fluorescence analysis (XRF) method (Figure S4), confirming the uniform distribution of elements. The X-ray diffraction (XRD) results of the material after conducting stability tests and relithiation/delithiation cycles showed a slight increase in the halo, indicating a minor increase in disorder within the material (Figure S6). However, overall, the reflection angles did not exhibit significant changes in the crystal lattice of the material.

**Figure 11:**
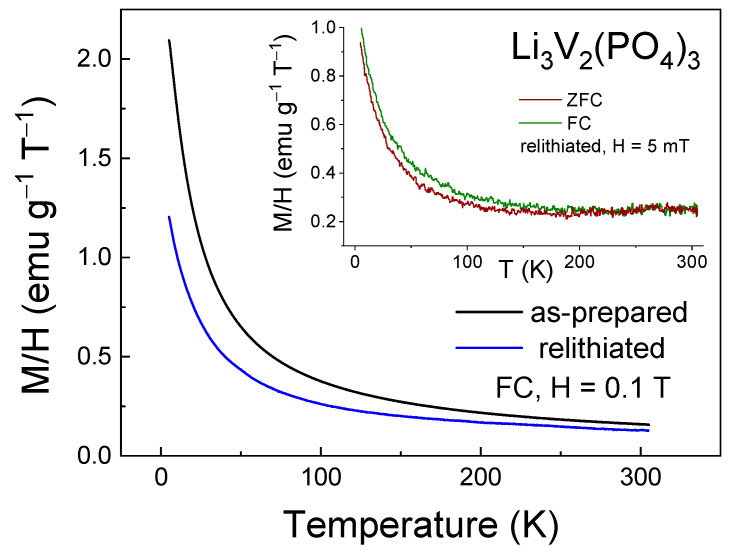
Magnetization M/H as a function of temperature (M-T curve) for as-prepared and relithiated Li_3_V_2_(PO_4_)_3_ samples measured in the FC regime in the external magnetic field of H = 0.1 T. Inset shows M-T curve for relithiated Li_3_V_2_(PO_4_)_3_ sample measured in the FC-ZFC in the external magnetic field of H = 5 mT.

**Figure 12:**
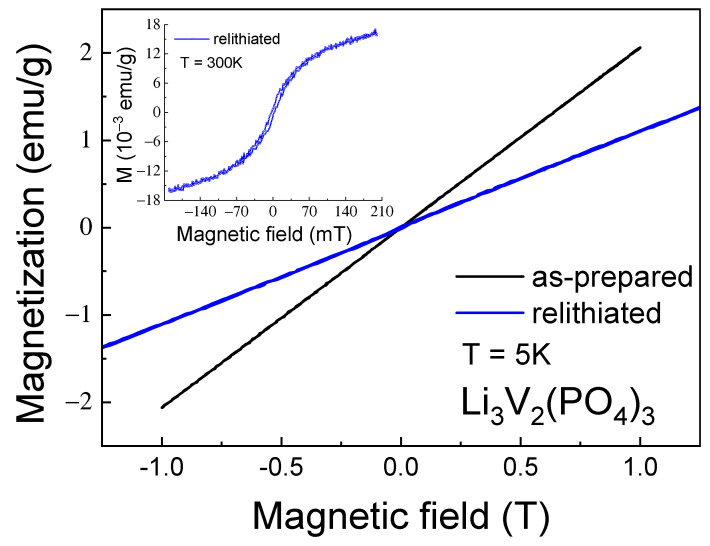
Magnetization M as a function of the external magnetic field (M-H curve) for as-prepared and relithiated Li_3_V_2_(PO_4_)_3_ samples measured at the temperature of T = 5 K. Inset shows the M-H curve for relithiated Li_3_V_2_(PO_4_)_3_ sample at room temperature.

The change in the magnetic properties of the multiple relithiated samples relative to the as-prepared one was detected by the magnetometry method, which is shown in Figure 11 and Figure 12. Noticeable differences are observed in both temperature dependences (Figure 11) and in the field dependences of magnetization (Figure 12).

**Figure 13:**
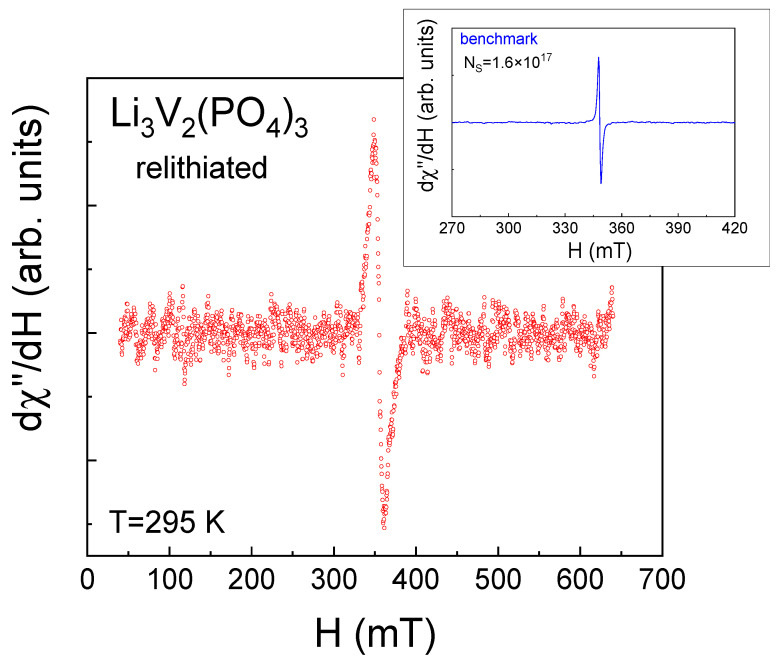
Electron resonance spectrum of relithiated Li_3_V_2_(PO_4_)_3_ at room temperature at the X-band frequency. Inset shows the electron spin resonance spectrum of the benchmark containing N_s_ = 1.6∙× 10^17^ spins.

Additionally, a quantification of the number of tetravalent vanadium ions was carried out using the ESR method, following the previously applied method for the as-prepared sample. It can be observed that the ESR spectrum of the studied sample (Figure 13) is more distinctly registered compared to that of the initial sample (as shown in Figure 3). This observation indicates an increased number of magnetic centers actively participating in the absorption of the radiofrequency signal. According to quantitative assessments of the spectrum, the concentration of tetravalent vanadium ions is approximately 7.5 percent. This substantial percentage of tetravalent vanadium ions is significant as it may be the cause of magnetic inhomogeneity within the sample. In other words, this high concentration of ions could lead to the formation of magnetically correlated regions. This phenomenon of magnetically correlated areas is something we, indeed, observed in our experiments focused on measuring magnetization. In essence, the enhanced intensity of the ESR spectrum in the multiple relithiated samples suggests a notable alteration in its magnetic properties, particularly in terms of the number and distribution of magnetic centers. The presence of 7.5% tetravalent vanadium ions significantly influences the magnetic behavior of the material, potentially leading to the development of regions within the sample where magnetic properties are correlated or linked. This correlation has been substantiated through our dedicated magnetization measurement experiments, revealing new insights into the magnetic nature of the material.

It should be noted that the observing ESR signal directly proves the absence of a metal phase in a relithiated LVPO sample since the presence of any metal inclusions makes it impossible, or significantly complicates, to detect ESR under conventional experimental conditions.

## 3. Discussion

The main findings of this article are based on the results obtained from electrochemical studies, with a particular focus on highlighting the high lithium-ion diffusion coefficient and the material’s stability under multiple cycling conditions. Additionally, other methods used in this study support and visually demonstrate the high-performance characteristics of the material under investigation. The X-ray diffraction (XRD) results suggest that despite the electrochemical cycling of relithiation and delithiation, the crystal lattice of the material retains its stability, implying that lithium diffusion occurs without significant alteration of structural parameters. Thus, the diffusion coefficient can be assessed based on the assumption of crystal lattice stability, which is crucial for further understanding the mechanisms of lithium-ion conductivity in this material.

Previous research, such as [50], has shown a decrease in capacity and a drop in efficiency to 40 percent over 500 cycles. In [41], a significant reduction in capacity was observed as early as 30 cycles. In [39], cyclic voltammograms for carbon composites were provided, and similar measurements for LVP@M-101 composites at different scanning rates of 0.05–0.25 mVs^−1^ were presented in [45]. The SEM images reveal that the samples exhibit a more pronounced granular structure with smaller granule sizes compared to LVPO/LPO composites [13,33], synthesized using similar technology. It appears that the presence of salt does not significantly influence the absolute values of specific capacitance, even slightly enhancing it. However, the pure phase is characterized by remarkably high diffusion coefficient values, which could be critical for practical applications, potentially impacting the speed of battery charging and discharging processes.

The investigation of the magnetic properties using ESR and magnetometry reveals a high degree of stoichiometry and magnetic homogeneity in the initial sample, indicating the absence of magnetically correlated regions of significant size that could be detected during experiments. The high stoichiometry and magnetic uniformity of the pure sample are markedly different from the properties of previously studied LVPO/LPO composites. This distinction is reflected in the electrochemical properties, particularly in the lithium diffusion coefficient, which shows higher values for the pure sample compared to the composite. The surface analysis of the sample through scanning electron microscopy (SEM) after multiple cycles revealed that the granular structure of the sample is maintained but undergoes substantial changes during a single charge/discharge cycle. The surface of the lithiated sample is noticeably different from that of the delithiated one, a phenomenon frequently observed and reported in numerous studies (see Figure 10).

Significant changes in the magnetic properties can also be observed. In particular, Figure 12 and Figure 13 compare the M-T and M-H curves of the initial and lithiated samples, respectively. A noticeable decrease in the absolute magnetization values of the lithiated sample across the entire temperature range studied is evident. The most likely explanation for this observation is the presence of a greater number of tetravalent vanadium ions in the lithiated sample compared to the initial one. As previously mentioned, V^4+^ ions have an electron spin of S = 1/2, whereas V^3+^ ions have a spin of 1. Therefore, the valence change from 3+ to 4+ leads to a reduction in the spin of individual centers and, consequently, a decrease in the macroscopic magnetic moment, i.e., magnetization. Indirect confirmation of this hypothesis can be seen in the divergence of the FC-ZFC curves (inset in Figure 11) and the appearance of a hysteresis loop in the field dependence of magnetization of the lithiated sample (inset in Figure 12). This may be due to the presence of ferromagnetic or ferrimagnetic correlated regions arising from the presence of magnetic ions with mixed valency (V^3+^/V^4+^). Apparently, the process of intercalation/deintercalation during multiple cycling is not entirely reversible in terms of restoring the valence state of the vanadium ions. However, this does not lead to a deterioration of the electrochemical properties.

The core focus of our study is to elucidate the pivotal role of the synthesis technique in producing carbon-free LVPO and its significant impact on electrochemical performance. Our work demonstrates that the methodology of obtaining carbon-free LVPO is of paramount importance because the traditional synthesis methods for LVPO are shown to result in a significantly shorter lithiation–delithiation cycle life [51,52,53]. The emphasis on the innovative synthesis approach of carbon-free LVPO in our study is to highlight the enhanced electrochemical stability and performance derived from the improved structural integrity, which is substantiated by the powder diffraction and X-ray phase analysis data. These analyses provide crucial insights into the material’s crystal structure and phase purity, which are directly correlated with the observed electrochemical performance. The comprehensive analysis through powder diffraction and X-ray phase analysis already contributes significantly to the understanding of the material’s performance characteristics, underscoring the methodological advancements made in the synthesis of LVPO for improved electrochemical applications.

## 4. Materials and Methods

### 4.1. New Method for Sample Synthesis

**Figure 14:**
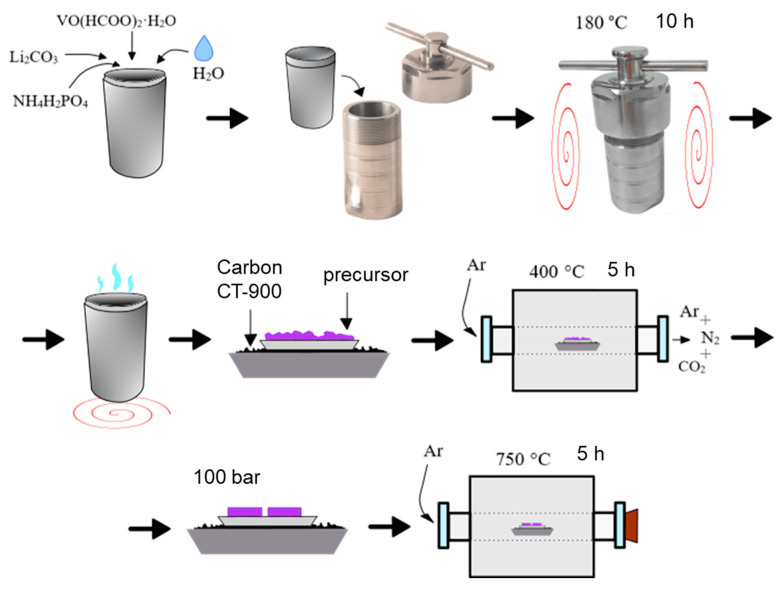
Hydrothermal synthesis scheme of Li_3_V_2_(PO_4_)_3_.

The pure Li_3_V_2_(PO_4_)_3_ (LVPO) investigated in this study was obtained via the hydrothermal method, followed by subsequent annealing in an Ar atmosphere. The synthesis was realized according to the scheme shown in Figure 14:

(i) Chemically pure vanadyl formate VO(HCOO)_2_·H_2_O, lithium carbonate Li_2_CO_3_ and ammonium dihydrogen phosphate NH_4_H_2_PO_4_ were used in stoichiometric molar ratios as starting materials.

Ammonium dihydrogen phosphate (NH_4_H_2_PO_4_) was selected as a reagent for the synthesis due to its amphoteric properties, chemical stability, high water solubility and potential to achieve the highest purity of product synthesis. Lithium carbonate (Li_2_CO_3_) was chosen for its electrochemical properties, high reactivity at elevated temperatures, low toxicity and safety during storage, low cost and widespread availability. It should be noted that lithium hydroxide LiOH, often used in similar synthesis processes, is hygroscopic, making it challenging to control the stoichiometry of the resulting reaction products.

Vanadyl formate (VO(HCOO)_2_·H_2_O) is a complex vanadium-containing salt of carboxylic (formic) acid and vanadyl ion VO^2+^. The vanadium ions in VO^2+^ are in a tetravalent state, a more reduced form compared to V_2_O_5_ or ammonium metavanadate NH_4_VO_3_. During the hydrothermal action inside the VO(HCOO)_2_·H_2_O molecule, the HCOO-VO-OOCH chemical bond is broken, and the 2HCOO^−^ anion decomposes into 2CO_2_ and H_2_ gaseous products. This provides a reducing atmosphere in the closed reactor and allows for a reduction in the temperature and time of hydrothermal treatment of the precursor. The synthesis details of vanadyl formate (VO(HCOO)_2_·H_2_O) are provided in the Supplementary Materials (Figures S1 and S2).

(ii) The reagents mentioned above were combined in an autoclave reactor (100 mL), using 5–6 mL of distilled water for homogenization. The autoclaving was conducted at 180 °C for 10 h, followed by cooling to room temperature without air exposure.

(iii) The resulting dark violet gel-like precursor was dried in an open Teflon glass in a drying oven until a constant weight was achieved (approximately 5 h). The dried precursor was then ground, pressed and subjected to carbothermal reduction: calcination at 400 °C in an argon flow (99.998%, “Linde”, TS 6-21-12-94) for 5 h in the presence of a carbon substrate (CT-900, Donkarb-Graphite). The carbon was placed in a separate crucible to prevent precursor contamination and the formation of by-products.

(iv) The resulting precursor was pressed at a pressure of 100 bar, placed onto a carbon substrate in a crucible and annealed at 750 °C for 5 h in a tube furnace within an inert atmosphere.

### 4.2. Structural and Microstructural Investigations Methods

The composition of the obtained Li_3_V_2_(PO_4_)_3_ (LVPO) samples was controlled using a Shimadzu XRD-7000 S automatic diffractometer (Shimadzu Corporation, Kyoto, Japan) (monochromatic Cu_kα_—radiation, λ = 1.54 Å) with 0.03° steps in the 10–70° range. An exposure of 2 s at a point was used. The phase analysis of the reaction products was performed using the crystallographic database “Database of Powder Standard–PDF2” (ICDD, USA, Release 2005). X-ray pattern processing was conducted according to the Rietveld method using the FULLPROF software (Version date 23.11.2023, https://www.ill.eu/sites/fullprof/php/downloads.html).

During the synthesis process, the phase composition of the final product was controlled using X-ray phase analysis with a temperature step of 50–100 °C. The resulting diffraction patterns are shown in Supplementary Materials (Figure S3). It can be observed that carbon starts to leave the system at 400 °C and is completely removed by 600 °C. The phase formation begins at 500 °C and is totally completed at 750 °C. An increase in temperature above 750 °C (up to 850 °C) does not change the phase composition but leads to agglomeration and partial melting of LVPO particles.

The surface morphology of the LVPO structure was analyzed using a Merlin scanning electron microscope (SEM) (Carl Zeiss AG, Oberkochen, Germany). The elemental composition and elemental mapping were investigated by the X-ray fluorescence analysis (XRF) method using Bruker M4 TORNADO micro-XRF Spectrometer (Bruker Corporation, Bremen, Germany).

### 4.3. Magnetic Property Investigation Methods

The electron spin resonance (ESR) spectra of the LVPO sample were measured using an ER 200 SRC (EMX/plus) spectrometer (Bruker Corporation, Germany) at a frequency of 9.4 GHz at room temperature. This was achieved using a double rectangular X-band resonator, ER 4105DR. This equipment enables the detection of the electron spin resonance spectrum of the investigated sample and the benchmark spectrum simultaneously.

The magnetization of LVPO was measured using a commercial PPMS-9 platform (Quantum Design, San Diego, CA, USA) in temperatures ranging from 5 to 305 K in field-cooled (FC) and zero field-cooled (ZFC) regimes. The magnetic hysteresis loops were measured in the magnetic field range of 1 T. The magnetization as a function of temperature (M-T curve) was measured in a magnetic field of H = 0.1 T in the FC regime and in a magnetic field of 5 mT in FC and ZFC regimes.

### 4.4. Electrochemical Property Investigation Methods

Electrochemical property investigations were carried out using the galvanostatic method in a three-electrode electrochemical cell with a PARSTAT 4000 galvanostat/potentiostat (AMETEK Scientific Instruments, Oak Ridge, TN, USA). The working electrode was a Li_3_V_2_(PO_4_)_3_ (LVPO) sample, while metallic Li plates were used as reference and counter electrodes. The used electrolyte was a solution of LiPF_6_ in ethylene carbonate (EC) and dimethyl carbonate (DMC), specifically a 1.0 M LiPF_6_ in an EC/DMC mix at a 50/50 volume ratio. All reagents were purchased from Sigma Aldrich and were of “Battery grade”. Electrochemical cell assembly was carried out in a glovebox under a dry argon atmosphere with oxygen content not exceeding 1 ppm. Galvanostatic cycling tests and cycle characteristics were studied within a voltage window of 2.5–4.5 V at room temperature. The mass loading value of the LVPO electrode is 7.4 mg/cm^2^.

Cyclic voltammetry (CV), commonly used in traditional electrochemical analysis to determine lithium diffusion coefficients, was applied to study the kinetics of ion diffusion in LVPO electrodes at scan rates ranging from 0.5 to 100 mV s^−1^. To calculate the diffusion coefficient for lithium ions in lithium vanadium phosphate (LVPO) or similar materials in electrochemical systems, the Randles–Sevcik equation is often used, especially in the context of cyclic voltammetry [18]. The Randles–Sevcik equation is given by

(3) $$Ip=(2.69\times 10^{5})n^{3/2}AD^{1/2}C_{Li}v^{1/2}$$

where Ip is the peak current in amperes (A), n is the number of electrons transferred in the redox event, A is the area of the electrode in square centimeters (cm^2^), D is the diffusion coefficient in square centimeters per second (cm^2^/s), $C_{Li}$ is the concentration of the reactive species in moles per cubic centimeter (mol/cm^3^), v is the scan rate in volts per second (V/s). This equation allows for the calculation of the diffusion coefficient (*D*) of lithium ions moving through the electrode material during the electrochemical reaction based on the observed peak current during a cyclic voltammetry scan. The equation highlights how the peak current is directly proportional to the square root of the scan rate and the diffusion coefficient, providing a method to estimate *D* from experimental data.

## 5. Conclusions

In this study, Li_3_V_2_(PO_4_)_3_ cathodes, synthesized via a hydrothermal method and further treated in an argon atmosphere, demonstrated promising characteristics for use in lithium-ion batteries (LIBs). The synthesis process ensured a single-phase material, as verified through X-ray diffraction analysis. The resulting granular structure, observed via scanning electron microscopy, suggested an advantageous uniformity in particle size, which is beneficial for the electrochemical functionality of these cathodes. Detailed magnetic analyses, including magnetometry and electron spin resonance, indicated the presence of tetravalent vanadium ions (V^4+^), a key factor influencing the cathode’s electrochemical traits. This particular valence state implied a lithium deficit in the initial structure, though the concentration of V^4+^ ions remained relatively low and evenly distributed across the material. This uniform distribution, evidenced by consistent magnetic properties, is crucial for reliable electrochemical performance.

For the synthesized pure phase Li_3_V_2_(PO_4_)_3_ sample, free from contaminants like carbon or salts, thorough electrochemical testing revealed high lithium diffusion coefficients, signifying exceptional ionic conductivity vital for LIB performance. The material also displayed outstanding stability over 1000 charge–discharge cycles, indicating its suitability for prolonged use in LIBs. The combination of these high lithium diffusion rates and sustained cycle stability underlines the potential of Li_3_V_2_(PO_4_)_3_ as a viable material for advanced energy storage solutions. Further investigations on the relithiated Li_3_V_2_(PO_4_)_3_ sample post multiple cycling, using methods such as scanning electron microscopy, magnetometry and electron spin resonance, were instrumental in assessing any material degradation.

## 6. Patents

The herein presented new method for sample synthesis is protected by patents: patent RU 2801381 C1, 8 August 2023, Synthesis Method of Cathode Material with the Composition Li_3_V_2_(PO_4_)_3_, Gyrdasova O.I., Deeva Yu.A., Chupakhina T.I., Gavrilova T.P., Khantimerov S.M.; patent RU 2732254 C1, 14 September 2020, Synthesis method of vanadyl (IV) formate (variants), Gyrdasova O.I., Krasil’nikov V.N.

## Figures and Tables

**Table 1:** ESR spectra integral intensity ratio I_LVPO_/I_0_, number of magnetic centers N(V^4+^), total number of vanadium ions N_0_, relative number of tetravalent vanadium ions and lithium deficiency for Li_3_V_2_(PO_4_)_3_ sample.

No.	Sample	LVPO
1	mass (mg)	7.4
2	I_LVPO/LPO_/I_0_	4.02/1.86
3	N(V^4+^)	3 × 10^17^
4	N_0_	21.79 × 10^18^
5	N(V^4+^)/N_0_	1.4%
6	lithium deficiency	0.9%

## Data Availability

Data are contained within the article and Supplementary Materials.

## Supplementary Materials

The supporting information can be downloaded at: https://www.mdpi.com/article/10.3390/ijms25052884/s1. Reference [54] is cited in the Supplementary Materials.
