# Supplementary material for: Li3V2(PO4)3 Cathode Material: Synthesis Method, High Lithium Diffusion Coefficient and Magnetic Inhomogeneity
Source: Int J Mol Sci. 2024 Mar 1;25(5):2884. doi: 10.3390/ijms25052884 (PMC10932188; doi:10.3390/ijms25052884)
Supplement: Supplementary file 1 [file ijms-25-02884-s001.zip › ijms-2856888-supplementary.pdf]

$Li_3V_2(PO_4)_3$ Cathode Material: Synthesis Method, High Lithium Diffusion Coefficient and Magnetic Inhomogeneity

Tatiana Gavrilova [1,*], Yulia Deeva [2], Anastasiya Uporova [2], Tatiana Chupakhina [2], Ivan Yatsyk [1], Alexey Rogov [1,3], Mikhail Cherosov [3], Ruslan Batulin [3], Mikhail Khrizanforov [4,5] and Sergey Khantimerov [1]

[1] Kazan E. K. Zavoisky Physical-Technical Institute, FRC Kazan Scientific Center of RAS, Sibirsky Tract, 10/7, 420029 Kazan, Russia; i.yatzyk@gmail.com (I.Y.); alexeyrogov111@gmail.com (A.R.); khan-tim@mail.ru (S.K.)
[2] Institute of Solid State Chemistry of the Ural Branch of RAS, Pervomaiskaya Str., 91, 620990 Ekaterinburg, Russia; juliahik@mail.ru (Y.D.); nastyauporova99@gmail.com (A.U.); chupakhina@yandex.ru (T.C.)
[3] Institute of Physics, Kazan Federal University, Kremlyovskaya Str., 18, 420008 Kazan, Russia; mcherosov@gmail.com (M.C.); tokamak@yandex.ru (R.B.)
[4] Arbuzov Institute of Organic and Physical Chemistry, FRC Kazan Scientific Center of RAS, Arbuzov Str., 8, 420088 Kazan, Russia; khrizanforov@gmail.com
[5] Aleksander Butlerov Institute of Chemistry, Kazan Federal University, 1/29 Lobachevskogo Str., 420008 Kazan, Russia
* Correspondence: t.gavrilova@knc.ru/tatyana.gavrilova@gmail.com

Supplementary Materials

S1. Vanadyl Formate Synthesis

The vanadyl formate synthesis was carried out according to the method described in [54] and showed in Figure S1:

✓ Vanadyl sulfate powder $VO(SO_4)_2{\cdot}3H_2O$ in an amount of 5 g is dissolved in 100 mL of distilled water.

✓ 90 mL of 12% NH_4OH solution is added to the resulting solution.

✓ The resulting brown oxide precipitate is filtered from the mother liquor using a filter paper with a pore size of 2–3 μm and clean with distilled water until there is no qualitative reaction to SO_4^{2-} ions (on $BaCl_2$).

✓ The precipitate is transferred to a heat-resistant glass with a volume of 600 ml, after that 100 ml of distilled water and 5 ml of 99.7% formic acid is added. In this case, the ratio of components is following vanadyl sulfate:ammonia (12–14%):formic acid (45–99.7%) = 1:2.0:1.5. After dissolving the brown precipitate, a blue solution is formed, which is evaporated at a temperature of 50°C for 90 minutes until vanadyl formate crystallizes.

✓ The X-ray phase analysis of the dried to constant weight blue crystals showed the resulting product is the single phase sample (Figure S2) with the chemical formula $VO(HCOO)_2{\cdot}H_2O$).

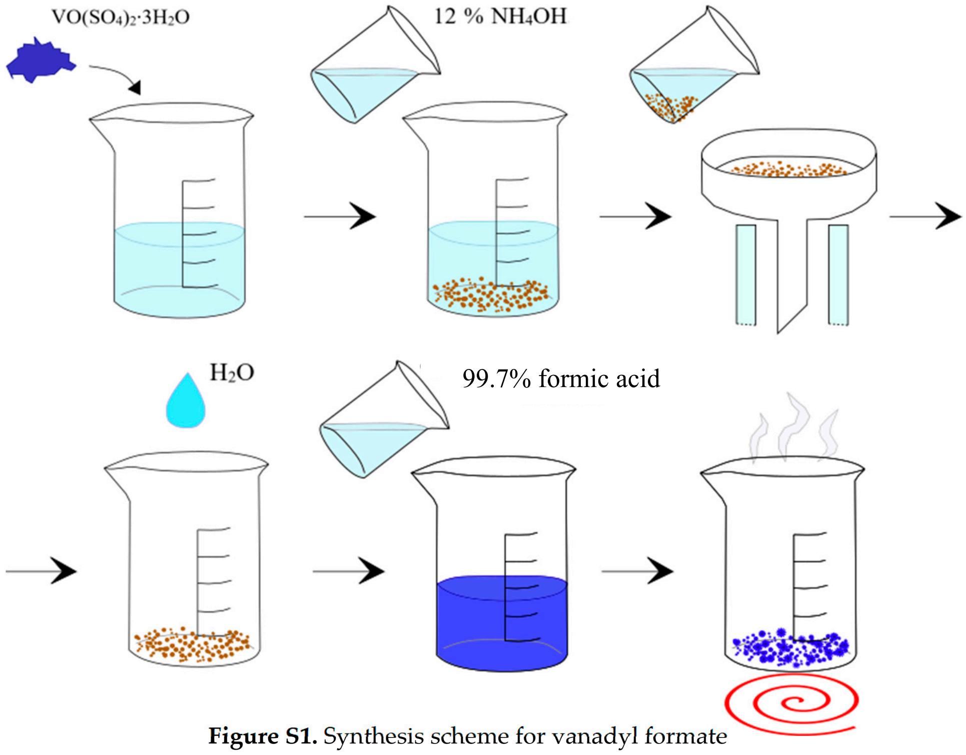

Figure S1. Synthesis scheme for vanadyl formate

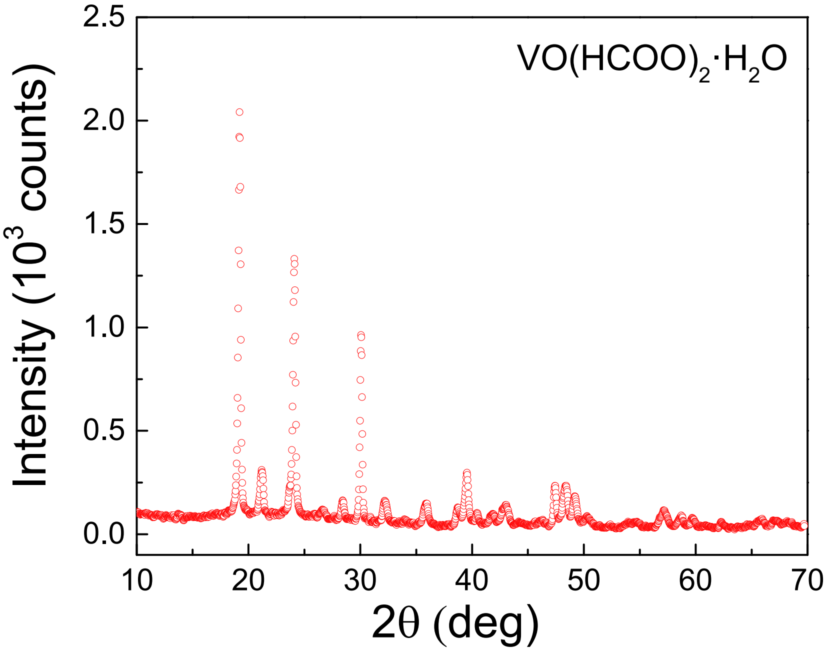

Figure S2. X-ray diffraction pattern of $VO(HCOO)_2{\cdot}H_2O$

S2. Phase formation of $Li_3V_2(PO_4)_3$ during the heat treatment

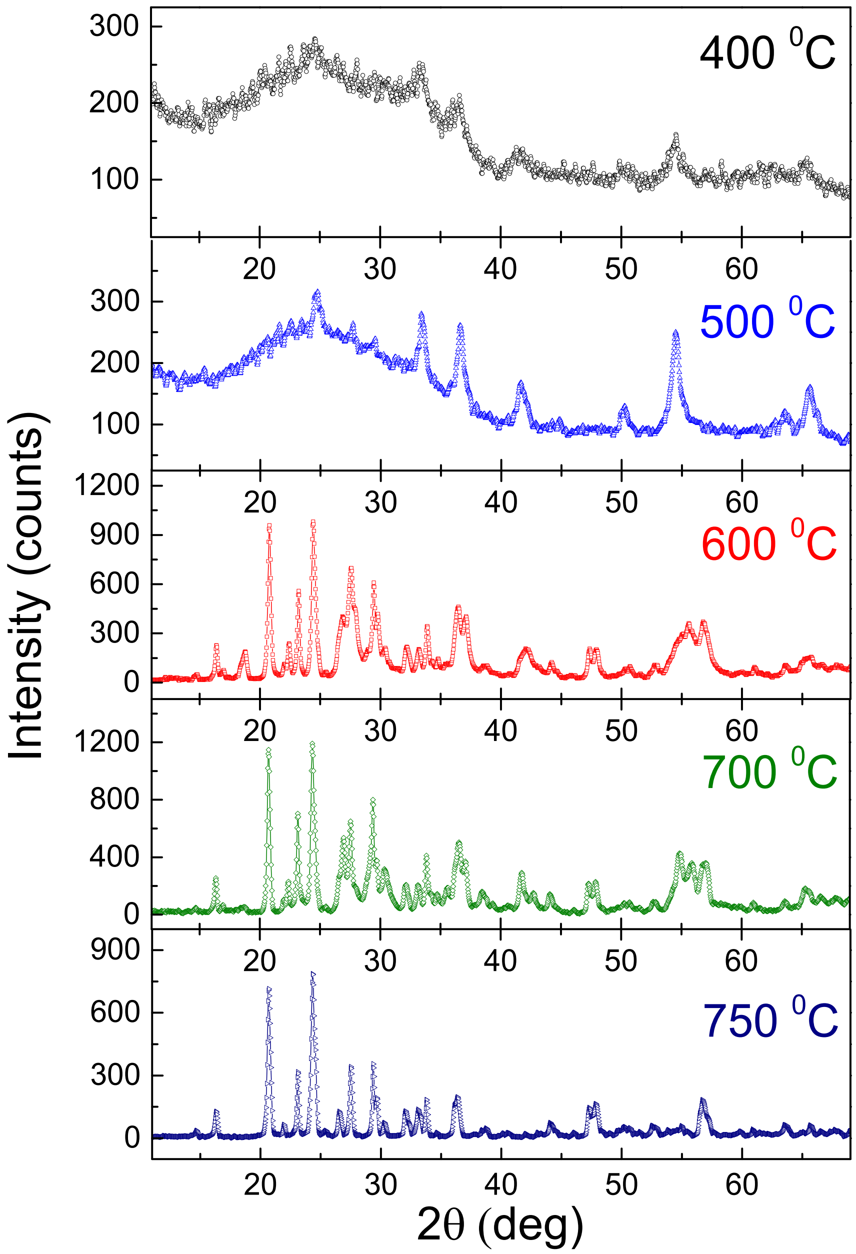

Figure S3. X-ray diffraction patterns of the precursor after thermal hydrolysis during the phase formation process of $Li_3V_2(PO_4)_3$ during sequential heat treatment up to 750 ^{0}C

S3. X-ray fluorescence analysis

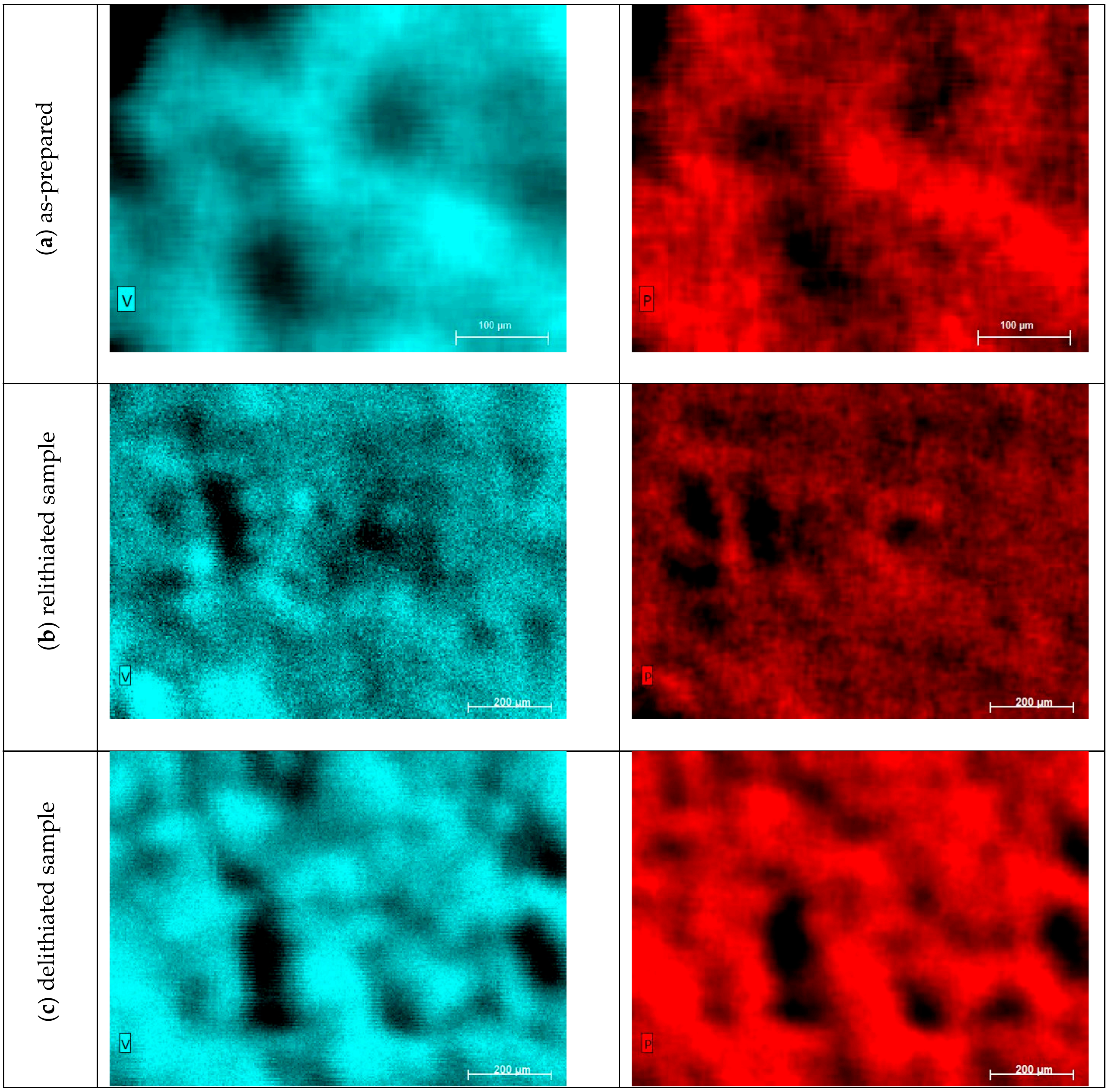

Figure S4. XFA images of LVPO (**a**) as-prepared sample, (**b**) relithiated sample and (**c**) delithiated sample. Dark areas correspond to the absence of a sample

S4. Magnetization measurements of $Li_3V_2(PO_4)_3$-based composites

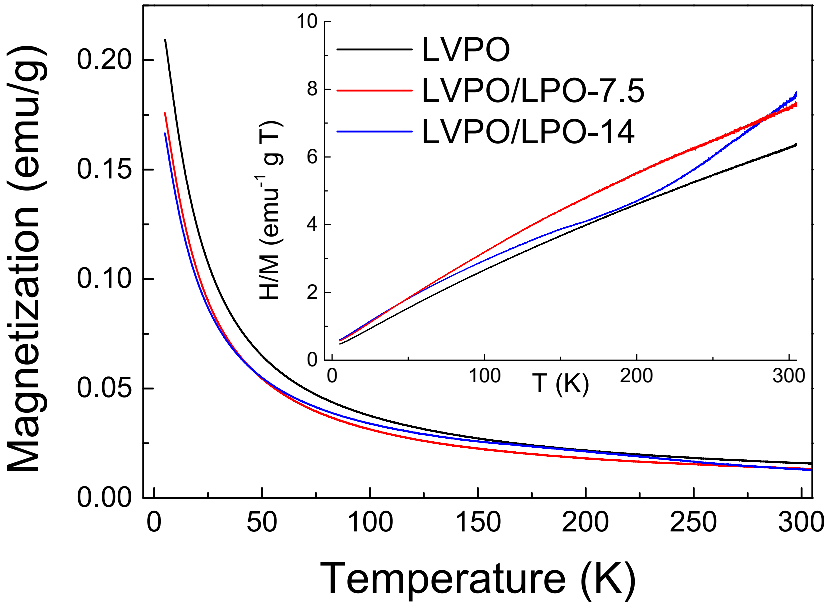

Figure S5. Magnetization M/H as a function of temperature for $Li_3V_2(PO_4)_3$ sample and $Li_3V_2(PO_4)_3$ (86 wt.%) / Li_3PO_4 (14 wt.%) and $Li_3V_2(PO_4)_3$ (92.5 wt.%) / Li_3PO_4 (7.5 wt.%) composites measured in the FC regime in the external magnetic field H = 0.1 T. The composites $Li_3V_2(PO_4)_3$ (86 wt.%) / Li_3PO_4 (14 wt.%) and $Li_3V_2(PO_4)_3$ (92.5 wt.%) / Li_3PO_4 (7.5 wt.%) are labeled as LVPO/LPO-14 and LVPO/LPO-7.5, respectively. Inset shows the inverse magnetic susceptibility H/M as a function of temperature.

S5. X-ray diffraction analysis

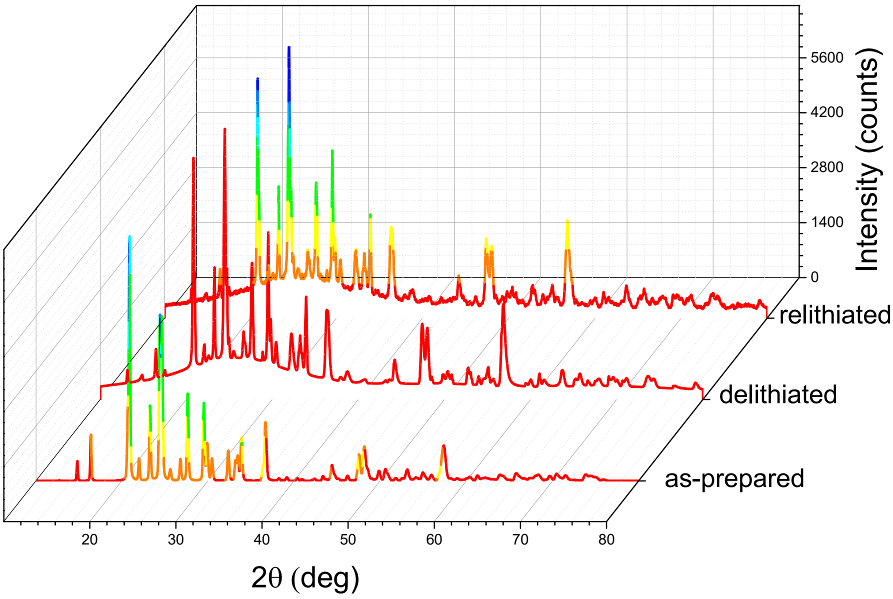

Figure S6. Experimental X-ray diffraction pattern of $Li_3V_2(PO_4)_3$: as-prepared, delithiated and relithiated samples.
